# A Psychosocial Approach to Understanding Underground Spaces

**DOI:** 10.3389/fpsyg.2017.00452

**Published:** 2017-03-28

**Authors:** Eun H. Lee, George I. Christopoulos, Kian W. Kwok, Adam C. Roberts, Chee-Kiong Soh

**Affiliations:** ^1^School of Civil and Environmental Engineering, Nanyang Technological UniversitySingapore, Singapore; ^2^Decision, Environmental and Organizational Neuroscience Lab, Nanyang Business School, Nanyang Technological UniversitySingapore, Singapore; ^3^Culture Science Institute, Nanyang Business School, Nanyang Technological UniversitySingapore, Singapore; ^4^School of Humanities and Social Sciences, Nanyang Technological UniversitySingapore, Singapore

**Keywords:** underground space, social factors, cultural psychology, community, built environment, urbanization, sustainability

## Abstract

With a growing need for usable land in urban areas, subterranean development has been gaining attention. While construction of large underground complexes is not a new concept, our understanding of various socio-cultural aspects of staying underground is still at a premature stage. With projected emergence of underground built environments, future populations may spend much more of their working, transit, and recreational time in underground spaces. Therefore, it is essential to understand the challenges and advantages that such environments have to improve the future welfare of users of underground spaces. The current paper discusses various psycho-social aspects of underground spaces, the impact they can have on the culture shared among the occupants, and possible solutions to overcome some of these challenges.

## Introduction

While urbanization is becoming a global trend (Cox, 2015), sustainability of the city environment is becoming a growing concern. A critical factor to be addressed is increasing population density, which is closely related to deterioration of the quality of urban life. This has led to a typical scene of cities in which high-rise buildings are abundant. However, with limited capacity to build skyscrapers the necessity to develop underground complexes that can accommodate the needs of a large population has become more apparent (Li et al., 2016). Asia, in particular, is adopting this solution. Beijing is constructing three million square meters of underground space each year (Chen et al., 2014). It is estimated that Guangzhou will have 5 m^2^ of underground space per capita by 2020 (Gao and Li, 2014). Singapore has started an underground master planning taskforce to identify different underground space uses (Zhou and Zhao, 2016). Likewise, Seoul recently announced its proposal of building a large scale underground complex that extends several kilometers, linking 12 metro stations underground (Lee J., 2016).

Although the concept of underground urban areas is not new (e.g., the underground city network RÉSO in Canada), our understanding of the social environment of subterranean structures is still at an immature stage. Most existing underground buildings are transitory spaces (e.g., shopping malls, underground subway stations) but with projected growth of subterranean buildings it is important to set up a framework for developing better underground spaces.

The aim of the present paper is to discuss potential problems associated with underground spaces. Besides technological aspects, psycho-social factors could affect willingness to engage with underground spaces (Soh et al., 2016). The literature on this topic is rather sparse and many studies are over 20 years old, using interviews and questionnaire measures (e.g., Hollon et al., 1980; Carmody and Sterling, 1987, 1993; Nagy et al., 1995; Küller and Wetterberg, 1996). While additional research using newer methods is needed, we can use the common themes from past studies to inform this research. Through a literature review of core texts in this area, we identified four major issues: isolation, perceived control, negative culture-based associations, and perceived security. This paper attempts to provide a critical review of these psychological phenomena and how they contribute to the culture of future subterranean environments.

## General Perspectives on Underground Space

An underground space is typically known as an enclosed environment below the surface of the earth. This means that unlike aboveground built environments, there is no direct access to outdoor open spaces (Ringstad, 1994). Thus, users of underground spaces do not get a straight view of on-going events outdoors. Often, this feeling of entrapment is associated with loss of control over the environment which can cause uneasiness and claustrophobic reactions (Hane et al., 1991; Ringstad, 1994). Moreover, due to the absence of sunlight and natural scenery, the space tends to be darker without much variation throughout the day. This lack of stimulation seems to result in disfavor of underground spaces (Hane et al., 1991).

There are various societal beliefs about underground spaces, stemming from ancient times. For instance, Christianity depicts hell as the world underground (Lesser, 1987). Likewise, Taoism and Buddhism relate underground to the concept of ‘diyu’ [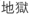], the realm of the dead in Chinese cultural beliefs. In addition to these religious beliefs, human burial practice is common in both Eastern and Western society. This results in the impression that staying underground is like being buried, which adds to the association that underground represents death (Sommer, 1974; Hane et al., 1991). Overall, there seem to be many negative connotations of underground space across cultures.

While there are negative conceptions, there are also positive aspects of underground spaces. For instance, underground city RÉSO, the large underground complex linking various commercial and office buildings, has over half a million visitors during the long winters of Montreal. Similarly, underground malls in Singapore are used as alternative recreational and social spaces to escape the tropical climate (Kong, 2013). Underground spaces provide safety during war or other major crises (Mohirta, 2012). Underground space also has its benefit in energy efficiency as the ground functions as a thermal reservoir for interior temperatures, reducing the use of fossil fuels (Ma et al., 2009). In addition, people from different countries view the underground environment with diverse opinions, such as Americans being more likely to associate an underground space with comfort than the Japanese (Hane et al., 1991). Further, employees with experience of working underground are more positive about working underground in the future (Carmody and Sterling, 1990). This implies that perception of underground structures may depend on the culture, environment, and experience people have.

## Psychosocial Characteristics of the Underground Environment and Cultural Development

The unique underground environment could result in specific psychosocial characteristics, but these do not necessarily result in adverse effects on the culture of the community. The atypical environment of underground space may promote cooperation among people and drive the community toward being more collectivistic – in many ways, culture and the sense of community could develop when there is higher uncertainty (Christopoulos and Hong, 2013; Christopoulos and Tobler, 2016), whereas cultural symbols and landmarks could reduce stress (Yap et al., 2017). The challenge for any future underground development is to identify these characteristics, recognize their effects on the community, and design the environment or the work practices to take these into account. For example, cooperation can be fostered within a company in different ways to take into account individualism or collectivism (Chen et al., 1998). In the current section, we discuss what these characteristics are, how they may impact the culture shared among the users of underground spaces, and potential interventions that could be employed to overcome these characteristics (see **Table 1** for a summary).

**Table 1:** Potential issues and possible solutions.

Issue	Solutions
Isolation	• Additional transit connections
	• Introduction of natural light
	• Intermediary spaces
Lack of control	• Enhanced landmarks
	• Greenery
Negative associations	• Emphasis on privacy and safety
	• Increased high-end uses
Perceived security	• Added surveillance
	• Improved visibility

### Isolation

Underground structures have limited access to outside, meaning that in some way people are isolated. Indeed, a feeling of isolation from aboveground is consistently reported as one of the psychological constructs associated with underground (Sommer, 1974; Hollon et al., 1980; Vaught and Smith, 1980; Wada and Sakugawa, 1990; Ringstad, 1994). If the perception of such a barrier between above- and underground can be reduced, there should be less reluctance to join an underground “community.” Construction of more exit routes to aboveground could be a helpful architectural intervention (Carmody and Sterling, 1987). According to ecological psychologists (Gibson, 1979), a physical environment that affords certain behavior changes how one perceives the environment accordingly. In other words, if there is an increase in the number of elevators and escalators connecting to the ground level, traveling between these floors may seem less effortful, hence, less isolated from aboveground. Similarly, construction of light wells or skylights may further increase feelings of connection to the outdoors by letting natural light in to the underground structures.

Development of an intermediary space between under- and aboveground could be another solution to reduce the perceived barrier. For instance, a low-sloped passageway that connects the underground space to ground level, removing the obvious floor difference, could help to reduce the awareness of separation between under- and aboveground. Such a passage may have offices and various facilities so that it does not only function as a passageway but as a utilitarian space. Likewise, construction of large subterranean passages that mimic streets aboveground can promote a sense of familiarity from users. Increasing the usage of underground streets will decrease the number of times they travel up to exit the underground buildings. Such interventions could prevent awareness of being underground.

On a different note, this sense of isolation can function as a bonding agency. For instance, as people start identifying themselves as a member of a specific group – the underground community – individuals’ sense of belonging to the group is enhanced (Field et al., 1957; Tajfel and Turner, 1979; Vaught et al., 2000). Indeed, Vaught and Smith (1980) found that social solidarity and cohesion are critical factors of the culture shared among coal miners and that negative affects coming from the isolated environment and the nature of work were alleviated through social cohesion. Such a social environment promotes collectivistic culture. Cultural psychologists view cultures as being largely positioned across the collectivism/individualism dimension: Collectivistic cultures focus on group goals and value cooperation among people of the group, whereas individualistic cultures tend to orient themselves around self rather than a group (Hofstede, 1980; Oyserman et al., 2002; Oyserman and Lee, 2008). According to Social Identity Theory (Tajfel and Turner, 1979), a threat to a positive social identity may result in accentuation of positively valued differences and stronger in-group identification (Ellemers et al., 1999). For instance, ethnic minorities stress the self-defining values and importance of their ethnic background when they face negative characterisations of their group. Further, such emphasis can be coupled with feelings of pride and contentment regarding their ethnic identity (Verkuyten, 1999). This suggests that the people of the underground community may have accentuated in-group identification, which, in turn, contributes toward preservation of the culture shared among the community.

Based on this evidence, heightened social identity among the underground community may serve as an essential protective mechanism for the people (Vaught, 1991; Lee et al., 2016) and create a society in which collectivistic culture is shared. Thus, it would be important to assist their need for socializing. Creating an active socializing spot, which promotes interaction among underground users, would aid the required social support. A large area could be dedicated for facilities that could accommodate various underground users to come together and spend their recreational time. These facilities could include restaurants, an indoor park or communal gym, which could naturally bring the underground population together and enhance interaction among them.

### Lack of Perceived Sense of Control

The underground environment limits the capacity of various actions that could be performed, such as opening windows to ventilate the room or adjusting blinds to control the natural light in the space. Such circumstances could contribute toward a lack of perceived control. Perceived control is a critical construct in psychology which can influence both mental and physical health (Bosma et al., 1999; Bailis et al., 2001; Lundberg et al., 2007). It reflects the extent to which an individual believes that a situation or one’s environment is controllable and that one can bring about desired outcomes (Smith et al., 1984).

Several features of underground structures further lower individuals’ perceived control (Carmody, 1997). One of the prominent architectural elements of underground spaces is lack of windows. A windowless environment limits the actual control we have over the room, but it also creates an illusion that we have even less control than we have. When people notice that there is no window in the room, they instinctively think that evacuation may be hindered (Fich et al., 2014). Fich et al. (2014) demonstrated that participants put in a windowless environment responded with pronounced cortisol reactivity (i.e., stress hormone) to stress induction compared to participants in an environment with a virtual window. Considering cortisol activity is a part of the stressor effector system that reacts to unescapable stress, the finding supports the notion that windows can enhance a sense of control. More importantly, even with a virtual window people felt much safer, implying that mere perception of windows can determine how occupants feel and behave.

Another problem that a windowless environment has is lack of landmarks. Obstructed navigation due to landmarks being occluded by walls and ceilings results in a lower sense of control (Ringstad, 1994; Yokoi et al., 2015). Moreover, static conditions such as similarity in lighting, interior design, and traffic organization throughout a building results in further deterioration in wayfinding (Hane et al., 1991; Lee et al., 2016; Roberts et al., 2016). Having a deficiency of environmental cues to locate oneself within the environment could be a serious problem in case of an emergency as time pressure and physical threat (e.g., fire) could induce a hypervigilant state in which individual’s capacity to process environmental information deteriorates even further (Ozel, 2001). Thus, the lack of exterior and interior environmental cues in underground spaces impairs navigation, which in turn results in a decrease in occupants’ perceived control.

Unfamiliarity stemming from a variety of technological aspects of an underground facility could cause a lack of confidence. Public perception of underground space tends to associate underground with private technical use, in comparison to aboveground which is seen as more public, open, and for general urban use (Labbé, 2016). It is more likely that the underground environment is fully surrounded by built structures with no natural features (Ringstad, 1994), and visibility of aboveground space, interconnection of spaces and visual contact with nature are all reduced underground (Zhao and Künzli, 2016). Such an unnatural setting may cause a feeling of foreignness, which further reduces a sense of control within the environment.

Similarly, lack of greenery has been identified as one of the problems of underground spaces. People generally prefer nature to built spaces, appreciating the intrinsic value of nature regardless of its functions for humans (Kaplan, 1983, 1993; Purcell et al., 1994; Howley, 2011). A large body of literature shows beneficial effects of nature on health, psychological well-being (Ulrich et al., 1991; Hartig, 1993) and satisfaction with life in general (Kaplan and Kaplan, 1989). Further, employees in a windowless office have been shown to personalize their workspace (thus, increasing their perceived control) by introducing plants and pictures of nature compared to employees in a windowed environment, exhibiting their yearning for nature (Bringslimark et al., 2011). By incorporating greenery into the design of underground structures, the loss of contact with nature can be compromised. Such a measure will promote the physical and psychological well-being of the underground community.

There are many factors of the underground environment that could reduce a sense of control. But the impact it has on the culture shared among underground users may not always be negative. When people have a depleted sense of control, there is a tendency for cooperation among the community to reduce uncertainty. People with a collective identification, thus, giving and receiving help, in an emergency situation have a greater chance of surviving (Vaught and Smith, 1980; Drury et al., 2009). Thus, the underground environment may again facilitate a collectivistic culture.

### Negative Culture-Based Associations

A typical underground environment is thought to have features that people are predisposed to fear. Further, subterranean spaces are associated with various negative cultural concepts. As mentioned earlier, in both Eastern and Western culture, the idea of underground is closely related to death and evil forces (Lesser, 1987; Wada and Sakugawa, 1990; Hane et al., 1991). Moreover, it has also been associated with cave societies or primitive cultures (Mohirta, 2012). In modern societies, basement spaces in cities sometimes provide a living space for those who are impoverished. For example, China, which is in the midst of urban revolution, has many migrants from rural areas living underneath the city of Beijing (Xinghua Net, 2012; Huang and Yi, 2015). The media refer to these basement tenants as ‘mouse tribe’ (shu zu), which depicts their poor standing. People easily associate the subterranean community with a particular cultural identity, which is often negative or underprivileged.

Re-conceptualisation of the underground community is necessary. One way to improve the perception is to broaden the usage of underground spaces while putting emphasis on the privacy and protection that they can offer. For example, COEX mall in Seoul has its reputation as being a multi-cultural spot with a variety of high-end facilities (i.e., aquarium, cinema, restaurants). The users enjoy the ease of accessing numerous amenities while valuing the protection the environment provides from harsh weather conditions and traffic congestion aboveground (Lee W., 2016). Similarly, some high-end basement facilities, such as luxurious bars or the sometimes extensive, quiet spaces underneath private mansions, provide good examples (Wilson, 2014; Daily Detroit, 2016; Webber and Burrows, 2016). While these places are equipped with prestigious amenities, the feelings of segregation and privacy that the underground space provides attract people who are eager to feel privileged. Increasing such uses may change the way the public views underground spaces and increase their willingness to join the community. Most importantly, the type of community (e.g., high-profile companies, shops, and facilities), comfort, and privacy that these spaces can offer should be highlighted when being introduced to the public instead of the concept of ‘underground’ on its own.

### Perceived Security: Hidden and Hiding Spaces

Security refers to risk or dangers stemming from human behavior (such as terrorist attacks, crimes, etc.); security should be differentiated from safety, which is more related to threats and risk stemming from the physical environment (accidents, health risks, natural catastrophes, etc.). While lack of landmarks can be uncomfortable for the general population, this may provide an opportunity for those with a criminal intent. Most crimes occur within an offender’s activity space, where there is no capable guardian (Cohen and Felson, 1979; Brantingham and Brantingham, 1995). When an underground space is characterized by many hidden spaces, it provides places to hide for those with criminal intentions while hindering navigation of those unfamiliar with the space, reducing the chance of offenders being caught. Thus, underground buildings could be a popular activity space for offenders with criminal intentions (Uittenbogaard and Ceccato, 2014). Similarly, the “basement scene” punk subculture uses underground spaces primarily to avoid the police (Lingel et al., 2012).

Although difficult wayfinding could provide opportunities for offenders, underground structures could actually be a safer place compared to other public places (La Vigne, 1997). The incorporation of surveillance within environmental design plays a key role in determining a potential offender’s likelihood of choosing the spot to commit crime (Clarke and Felson, 1993). For example, an investigation on light-rail stations in Los Angeles showed that there was an increase of crime rates for stations with dark/hiding places or poor visibility of the surroundings while the opposite pattern was detected for stations with improved visibility (Cozens et al., 2003). Increased surveillance and enhanced visibility of the built environment, compared to other open spaces, was shown to lead to a higher security level (Newman, 1972).

## Conclusion

As there is an expected increase of underground structures, more research on human-centered engineering is needed. It is especially important to pay attention to psycho-social factors associated with underground environments as more people are likely to work, shop, and commute in such spaces. The ways people think, feel, and behave are closely tied to individuals’ bodily interactions with the physical environment (Meier et al., 2012; Lee and Schnall, 2014). Thus, as new communities are formed in hitherto unfamiliar underground spaces, special care is needed to facilitate their transition and adjustment, especially by avoiding or ameliorating negative experiential factors. The current paper pinpoints possible issues regarding the subterranean environment and discusses how they can be improved. The psychosocial characteristics covered here show that underground spaces can result in both positive and negative effects, but negative feelings are often reported in studies of underground users. The challenge for any future subterranean community, therefore, is to reduce the negative association attached to underground structures so that the predisposition to avoid the space can be moderated.

## Author Contributions

EL, as the first and corresponding author, researched for the materials for the article and wrote up the manuscript. GC did major revision on psychological aspects of the article while KK focused on the cultural/social aspects of the article. AR revised and developed the social and psychological aspects of the article. C-KS provided valuable insight regarding underground engineering and urbanization and reviewed the article.

## Conflict of Interest Statement

The authors declare that the research was conducted in the absence of any commercial or financial relationships that could be construed as a potential conflict of interest.
